# Key informants’ perspectives on policy- and service-level challenges and opportunities for delivering adolescent and youth-friendly health services in public health facilities in a Nigerian setting

**DOI:** 10.1186/s12913-022-08860-z

**Published:** 2022-12-07

**Authors:** Olujide Arije, Tintswalo Hlungwani, Jason Madan

**Affiliations:** 1 Institute of Public Health, Obafemi Awolowo University, Ile-Ife, Nigeria; 2 School of Public Health, University of Witwatersrand, Johannesburg, South Africa; 3 Warwick Medical School, University of Warwick, Warwick, UK

**Keywords:** Family planning, Reproductive health, Sexual health, Adolescents and young people, Ogun state, Nigeria

## Abstract

**Background:**

Integrating the care of adolescents and young people into existing public health facilities requires deliberate efforts to address challenges related to policy and service provision. This study assessed key informants’ perspectives on policy- and service-level challenges, and opportunities, for implementing a strategic framework for adolescent and youth-friendly health services (AYFHS) in public health facilities in a Nigerian setting.

**Methods:**

Seventeen key informants were interviewed including members of the Adolescent sexual and reproductive health (ASRH) Technical Working Group (TWG), program managers of non-governmental organizations (NGO), State and local level health officials, and youth representatives, in Ogun State, Southwest Nigeria.

**Result:**

Findings from this study indicate that some health workers continue to have a negative attitude toward young people’s sexual and reproductive health. There was some level of inclusion of adolescents and young people living with disabilities in ASRH programming which is welcome and extremely important. Some of the challenges in ASRH service provision included insufficient coordination of activities of donors/partners working in the adolescent health space. Also found was the missed opportunity to strengthen policy implementation with research, and the need for increased focus on mental health, substance use, and other aspects of adolescent and young people’s health. There was noted the opportunity to explore the Basic Health Care Provisions Funds (BHCPF) as a new source of funding for health services for AYP in Nigeria.

**Conclusion:**

This study provided the context of the implementation of a strategic framework for adolescent reproductive health in a Nigerian setting from the perspectives of policy and service-level stakeholders. Opportunities for improving program delivery identified include ensuring research-based policy implementation and seeking program sustainability through tapping into new sources of funding.

## Introduction

The high benefit-to-cost ratio of investing in adolescent and young people’s health and wellbeing is well recognized globally [1, 2]. Many low and middle income countries have taken this cue by investing substantially in initiatives and interventions to promote the health of adolescents and young people (AYP) [3]. This includes development of national standards for adolescent health services following the launch of World Health Organization’s (WHO) *Global standards for quality of health-care services for adolescents* [4]. Despite this, adolescent and youth-friendly healthcare services (AYFHS) are still widely unavailable at primary and secondary healthcare levels in a country like Nigeria [5, 6]. Most services provided at these facilities are focused on care for the general population [7]. This situation is also observable in many high, middle, and low-income countries [8]. Integrating care of adolescents into existing public health facilities requires deliberate efforts. According to Goicolea et al [9], an AYFHS that will be successful requires legitimacy, the self-confidence of the implementers in trying new things, a transformative process, and an integral approach to provision of services to adolescents while ensuring that contextual factors at the national, local, and institutional levels are adequately catered for.

Barriers to the provision of optimal health services to adolescents and young people include structural, sociocultural and individual barriers [10]. The structural barriers include laws and policies that limit access to services, non-availability of infrastructure, and service characteristics such as long waiting time, inconvenient hours, lack of commodities, cost of services, and lack of privacy and confidentiality. Closely associated to these are sociocultural factors that drive negative attitudes of health workers toward providing AYFHS, including restrictive norms and stigma concerning adolescents’ sexuality. The situation is further compounded by the low competency of health workers to provide optimal adolescent health services as well as a lack of synchronization of adolescents’ and young people’s wants with policy priorities [11, 12]. Individual barriers occur at the level of the adolescents including incorrect knowledge (including myths and misconceptions), limited self-efficacy and individual agency, and lack of access to correct information, among others [10]. In order to make significant progress in assuring optimum health for AYP, the key policy, programmatic and service-level stakeholders at both national and sub-national levels need to understand adolescent-specific issues [13]. This will enable them to run a system that has addressed values and morals that may conflict with providing care services to adolescents and young people [14], and be able to assure service recipients of confidentiality and privacy [8, 15].

To integrate AYFHS into the public health facilities in Nigeria, the national government developed the *National guideline for integrating adolescent and youth-friendly services into Primary Health Facilities (PHC) in Nigeria* in 2015 and the revised *National Standard and Minimum Package for Adolescent Healthcare* in 2018 [16]. The recommendation in the National Standard document for applying the national standards at the sub-national levels includes, the commitment of the state and local government administrative leadership, the commitment of the local government health management team to provide supportive supervision, and the existence of an organization (non-governmental or community-based) or health-related training institution involved in providing health education and counseling services to adolescents.

**Fig. 1:**
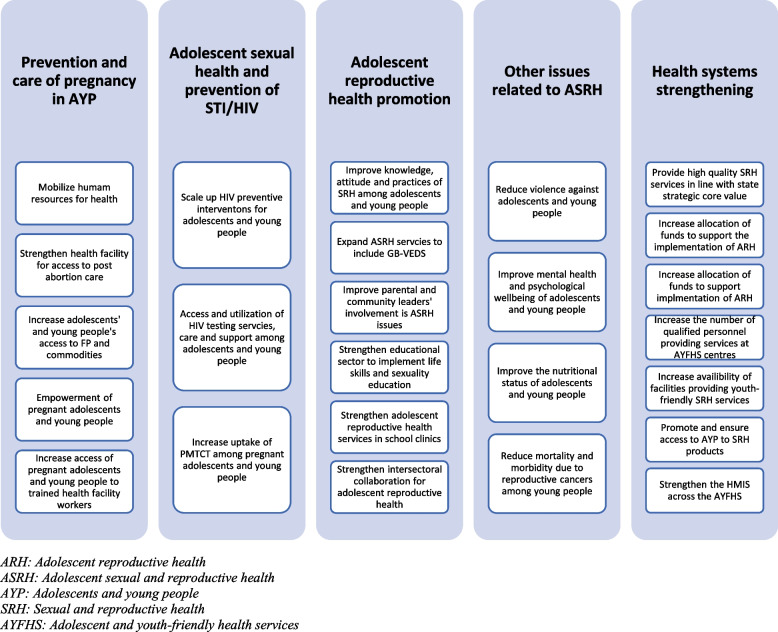
Priority areas and objectives of the Ogun State Adolescent Reproductive Health Strategic Framework (2018-2022)

Recently, Ogun State in Southwest Nigeria launched the Ogun State Adolescents and Young People’s Sexual and Reproductive Health Strategic Framework (2018 – 2022) document [17]. The framework covers strategic priority areas—including pregnancy prevention and care, the prevention of sexually transmitted infections, and health promotion—and defines each stakeholder’s roles and responsibilities (Fig. 1). This strategic framework is the first of its kind among the States in Nigeria, and puts Ogun State in a position to provide leadership and example in programming for AYP at the state level. The role of the policy-level participants and healthcare worker are critical in supporting this framework. Policy-level stakeholders for adolescent health include government officials from health-related ministries, departments, and agencies, as well as others from local and international non-governmental organizations, and the academia. Their activities help to facilitate an environment that gives a premium to the health of AYP. In the same manner, healthcare workers, who work at the frontline, are indispensable in revamping a health system that has been traditionally unfriendly to adolescents and young people, particularly as it relates to sexual and reproductive health.

With the launch of a strategic framework for adolescent reproductive health in Ogun State, the four mechanisms of Goicolea et al [9] for successful AYFHS are taking shape, starting with legitimacy through to ownership, grounded self-confidence in trying new things, the transformative process, and an integral approach to adolescents. In the wake of Ogun State’s implementation of its new framework, it is vital to study and understand some of the contextual factors that are likely to promote or prevent successful ASRH policy adoption and uptake. This research assesses key informants’ perspectives on policy and service-level challenges and opportunities for delivering AYFHS in public health facilities. Findings from this research will help to guide policymakers and program designers in addressing the factors associated with the provision of adolescent health services in the study area and beyond.

## Methodology

### Study design

This study has an exploratory qualitative research design using key informant interviews for data collection. The study took a phenomenological approach in order to be able to describe the experiences, events, or situations concerning the Ogun State Adolescents and Young People’s Sexual and Reproductive Health Strategic Framework (2018-2022) from the different perspectives of the participants.

### Study setting

The study location is Ogun State, which is in the Southwest geopolitical zone of Nigeria, and one of the 36 States in the country. The predominant language of the people of Ogun State is Yoruba, with the individual sub-ethnic groups of the state speaking different dialects [18, 19]. Ogun State has three senatorial districts that share a total of 20 Local Government Areas (LGAs) among them. Local Government Areas are divided into political units called wards, and Ogun State has 349 political wards in all. Adolescents and young people make up 30.7% of the state’s population [20]. According to the National HIV/AIDS Indicator and Impact Survey (NAIIS) of 2018, Ogun State had the highest HIV prevalence (1.6%) in the Southwest zone (zonal prevalence was 1.2%) [21]. In Ogun State, early sexual debut (at ten years) persists at 4.5% for boys and 5.3% girls, there is low comprehensive knowledge of adolescent reproductive health, and poor access to youth-friendly services among the young people [22].

### Sampling

Seventeen key informants were interviewed including members of the State’s ASRH Technical Working Group (TWG), program managers of non-governmental organisations (NGO) working in the adolescent reproductive health space in the state, key officials in the Ogun State Primary Healthcare Development Board (OGPHCDB), and key officials from two purposively selected LGAs (Abeokuta South and Ijebu East LGAs). The OGPHCDB is an agency under the Ministry of Health that is directly responsible for adolescent and reproductive health response within the state, and was the agency, in collaboration with Pathfinder International, that developed the Ogun State Adolescent Reproductive Health Strategic Framework (2018-2022) [22]. Abeokuta South LGA was purposively selected as a predominantly urban LGA, being the LGA that contains the State capital, while Ijebu-East LGA was selected as a predominantly rural LGA. These selections help to capture the spectrum of experience of across the State. One invited NGO participant declined to be interviewed due to organizational policy preventing them from some research involvement. Participants were recruited into the study based on their availability to be interviewed, and till key stakeholders at policy- and services-levels were fairly represented in the sample.

### Study instrument

The study instrument was a semi-structured interview guide that steered the exploration of participants’ opinions and perceptions about sexual and reproductive health services access in public health facilities within the state.

### Data collection

The key informant interviews were conducted by a fieldworker who has a postgraduate qualification in Public Health, and more than 8 years’ experience in conducting such interviews. Data was collected over 2 weeks in July and August 2021. The sessions were recorded using a digital recorder, and they held in the offices of the participants or other convenient and jointly agreed locations where privacy could be assured. Given the face-to-face nature of the interviews, COVID-19 prevention protocols were observed, including the use of facemasks, hand sanitizers, and physical distancing.

### Data management and analysis

All recorded sessions were transcribed verbatim. An initial code dictionary with thematic codes was generated based on the interview guide by OA, and revised by TH. The transcripts were subsequently coded to identify recurrent, dominant, and divergent opinions using the modified in-vivo coding approach [23]. In this approach, the opinions from the transcripts were organized in a hierarchy of abstraction of meaning, using codes, in which tertiary codes represented overarching thematic areas for the study. Secondary codes represented sub-categories within these overarching thematic areas, and the primary codes identified the contents of interest in the transcripts. The ATLAS.ti 9 software was used for creating the codes, attaching codes to data segments, and organizing and outputting quotations by themes and subthemes. Coding was done by an external qualitative data analyst with up to 5 years’ experience in coding transcripts in qualitative research. The coding was revised by OA. Synthesis of and ‘memoing’ from the analysis was done by OA and the external analyst. The findings were organized according to key themes that emerged from the coding process. The key themes that emerged included current government policies on ASRH, challenges in implementing existing ASRH policies and programs, gaps in current ASRH policies and activities, and opportunities for improvement of ASRH response in the study location.

### Ethics considerations

The study was approved by the Human Research Ethics Committee of the University of the Witwatersrand (#M210315) and the Ogun State Primary Health Care Development Board (OGHECADEB) Ethics Committee (#OGPHC/021/008). Written informed consent to participate and for audio-recording of the interview sessions was obtained from all participants. All methods in this study were performed in accordance with the Declaration of Helsinki [24]. We state the positionality of the authors of this research that: none of the authors has any direct or indirect affiliation with the government of Ogun State, Nigeria; and no government official or representative participated in the conduct, analysis or reporting of this research, apart from as participants in the key informant interviews.

## Result

Among the 17 key informants interviewed, five were State-level Ministry of Health officials, and five were LGA-level health officials (Table 1). There were two participants who were staff of NGOs operating within the state, two lay members of the ASRH TWG, and two youth representatives. Eight of the participants were males, while nine were females.

**Table 1:** Participants of key informant interviews

Type of participants	No.
State Ministry of Health Officials	5
State Official (Ministry of Women Affairs)	1
Local Government Health Officials	5
NGO	2
ASRH TWG Members	2
Youth representative	2
**Total**	**17**

### Current government policies and activities

#### Strategic framework for adolescent reproductive health

Some participants were of the opinion that the government of Ogun state currently had mechanisms in place to ensure ASRH services were accessible by AYP in the state. Current services and activities on adolescents’ reproductive health were said to target family planning, gender-based violence, and sexually transmitted infection. A member of the ASRH TWG said: *“…there is deliberate effort to have programs geared toward adolescent sexual and reproductive health, and this started with the inauguration of a technical working group on adolescent sexual and reproductive health, which I am also a part of. This acts like a policy component where we look at issues around adolescent health, and we are able to program for them accordingly,*…” [TWG member 1, M]. Similarly, an official of the Ministry of Health said: “*…a lot of efforts are going on, for example, we have a document that was specially designed for them, which is called the strategic framework document, and this takes care of all problems adolescents are facing within the State. The idea of the document was that for any partner coming, they will look into this document, not that they just come with their own plan or whatever…”* [Ministry of Health Official 1, F]*.* The framework was to be a guideline to be adopted by all stakeholders operating within the state in carrying out programs and interventions directed at AYP SRH. Riding on the success of the released strategic framework, a State Ministry of Health official said: *“… this policy has helped us to be able to have a room or to cater for our youths in Ogun state. Late last year we developed AOP (Annual Operational Plan) in which we decided to look into youths accessing some sexually transmitted infection (STI) drugs, which you know might help them; and might assist them to prevent sexually transmitted infection or to cure sexually transmitted infections…”* [Ministry of Health Official 2, M]*.* This annual plan was said to include training of health workers on adolescent reproductive health, provision of family planning services within facilities and as outreach services to AYP, and making provisions for including adolescents with disabilities in services provision.

#### Support for adolescent sexual and reproductive health

Most respondents indicated that there was now high-level support for ASRH services especially following the launch of the State’s adolescent reproductive health strategic framework. This includes the inauguration of a TWG with members comprising stakeholders from the Ministry of Health, related government ministries like Women Affairs, and Education, non-governmental organizations, civil society organizations, religious bodies, and youth representatives. All stakeholders actively engaged in ASRH in the State are obliged to attend the TWG meeting, where planning, synergizing, and coordination of ASRH for the state takes place. Also, health programming at policy-level relating to adolescent reproductive health within the State currently includes all relevant aspects of ASRH. According to a State Ministry of Health official: “*we look into various aspects; (including) …family planning, …adolescent and youth reproductive health, … gender-based violence … and sexually transmitted infection…”* [Ministry of Health Official 2, M]*.* A member of the ASRH TWG also commented that the State was also ready to adopt and adapt every other national policy on reproductive health and family planning.

#### Partnerships between government services and other organizations

Participants said non-governmental organizations and partners are engaged by the government in the joint implementation of programs on ASRH. Examples of programs carried out include skills acquisition programs for out-of-school youths, training of peer educators, and provision of family planning commodities. The partnership was also said to include an interfaith advocacy group that engages with religious leaders on the benefits of family planning and contraceptives. They speak to religious leaders on the issues of youth and adolescents accessing family planning. According to a member of the TWG:

*“…the state also has various advocacy groups among which are state advocacy working, group, then we have an interfaith alliance for family planning comprising the Imams and Pastors in the State, and also they develop a plan, and all of the plans also include the adolescent and youth reproductive health, where we go out to speak to religious leaders about the issue of youth and adolescent accessing family planning…” [TWG member 1, M].*

#### Engaging young people in planning and implementation

According to participants in this study, AYP representatives are engaged to participate in the planning and implementation of ASRH related programs and activities in the State. Firstly, the youth arm of the advocacy working group was very instrumental in pushing for the multi-sectoral strategic framework that would address the special needs of AYP. Now that the framework has been launched, they continue to be engaged as stakeholders in its implementation. A youth representative on the TWG commented: *“… we have these ambassadors in the advocacy working group, we have them in the technical working group and one of us even coaches the technical working group, … we help to implement policies, we help to tell them, oh this idea, is this what you want to be solved…”* [Youth representative 1, F].

#### ASRH programmatic activities

Some specific programmatic activities within the State include peer educators’ training and deployment of ‘life planning ambassadors’ who are saddled with sensitizing their peers in communities and schools. According to a State-level ministry of Health official “*presently in the state we have the adolescent, we have the life planning ambassadors. These life planning ambassadors are in different local governments in the State, these Life Planning Ambassadors are youths, they go out, talk to their peers, talk to people to out-of-school youths*…[and] … *give them the right information on sexual and reproductive health”* [Ministry of Health Official 3, F]*.* In addition to this, some participants agreed that there was an increase in the youth-friendliness of healthcare centres within the State. A youth reprsentative said: *“One major thing that I know that they have done is to integrate AYRH services to all existing PHCs; …in Ogun State we used to have two youth-friendly centers and those two youth-friendly centers would be where young people go and take up a* (family planning) *method, right, …they decided that instead of building youth centers lets integrate it so that anywhere you are living you can walk into any facility and receive a service.”* [Youth representative 1, F]. This was corroborated by a member of the ASRH TWG who said: “*Overtime too, we have … adolescents’ youth-friendly health services that are integrated into our Primary Health Care systems, where young people are expected to go there and also seek both preventive and curative care with respespect to their sexual and reproductive health”* [TWG member 2, M]. Furthermore, Ogun State was said to be actively promoting the access of AYP with physical disabilities to the right information, services, and care concerning sexual and reproductive health. A member of the TWG said: “…*for the physically challenged; in the state, there is a policy in the state, you don’t discriminate them, there is no discrimination against physically challenged youth; so, they also have access to reproductive health information and reproductive health services”* [TWG member 1, M].

It was mentioned that health workers trained in adolescent and young people’s SRH are being assigned to facilities to encourage the influx of AYP to access services at the facilities. Also, mentioned was that health workers are being trained to increase their knowledge of counselling and family planning. This is seen in the comment of one of the participants who said: *“…we just built the capacity of about fifty health workers on adolescents and youth reproductive health in the state. We are doing this because we know that most youths are unable to access health facilities because of the bias of the health worker, so, we are trying to put it up in a way that it will make the health worker see youths as very key to the development of the community, and the country as a whole.”* [Ministry of health official, 3, F]. Moreover, community campaigns and outreach programs are done to educate and sensitize people on sexual and reproductive health programs and available interventions by these health workers. However, there was an acknowledgment that more health workers need to be trained, according to a State Ministry of Health Official who noted that “*…we still need to carry out more training for some of them that are yet to be trained, not all were trained, but the majority have been trained”* [Ministry of Health Official 1, F]*.*

### Challenges in implementing existing reproductive health policies /activities

Funding was one of the challenges identified in the implementation of adolescent reproductive health policies within the state. According to a State Ministry of Health Official; “*… Ogun State is not having a purse to cover or to address…the plan* (strategic framework)*; we don’t have a designated purse to execute those plans, except if we have partners that come in to help”* [Ministry of Health Official 1, F]*.* Another challenge identified in the implementation of the ASRH policy was the negative attitude of some health workers. Several participants felt that some health workers believe adolescents are too young to use contraceptives. For instance, an NGO staff said *“…at the facility level that is health centers, the bias is still there, … I have seen it…the girl just came in …that… she wanted to take family planning, and the woman said, “how old are you? How many children do you have? You better go home and have a rethink of your life. What do you want* (from your life)*?”* [NGO Staff 1, F]. Such negative attitude was said to be associated with stigmatization, and has led to low patronage by AYP. Furthermore, participants commented that there was a shortage of staff as well as other resources to effectively provide services. Closely related to this is the issue of the shortage of medical supplies, consumables, and other materials needed to provide services. According to an NGO staff, *“…working with young people you need to be dynamic; you need to also spend resources which is usually not always there. …usually, most interventions cannot or do not have that kind of resources to engage”* [NGO Staff 2, M]*.*

Attitudes of young people to available services was another challenge that was identified. Young people are said not to willingly access services in public health facilities, preferring places that are not open to public view. Another take was that the SRH that focuses on adolescents and young people was still relatively new and many young people were skeptical about it. A male local government health official said: *“On the part of the adolescents, they’ve not embraced those programs and they are still skeptical maybe because much attention has not been given to them in the past”.* Also, some AYP were reported to object to the use of contraceptives because of concern about future fertility. One of the Local NGO staff said: *“So, most of them normally get scared saying: “ha, I have not given birth, o; how did you want me to do it”* (i.e.family planning)*”* [LGA health official, 2, M]*.*

A closely related challenge is community-level practices about family planning. Young people’s access to family planning and contraceptives is believed will lead to promiscuity and multiple sexual partnering. An example was a finding in which mothers-in-law were said to control the access of young married AYP to contraceptives. An LGA-level health official said: *“…mothers-in-law will not allow you to talk about family planning because they have the belief that if they* [i.e. their daughters-in-law] *engage in any method, it will make them promiscuous. …when you talk about family planning, they will say, “you want to engage them in this prostitution”; or they will say “…no, no, no, just go; don’t introduce our children to all these uncultured habits”* [LGA health official 1, F]. It is worthy to note an ongoing intervention that seeks to incorporate parents into an AYP intervention. A State-level Ministry of Health official said: *we still have issues with, the poor involvement of the parents too. Its only for the facilities that we have A360* [ongoing ASRH intervention] *that we have moms’ section…”* [Ministry of Health Official 4, F]*.* Also, one participant felt that some religious leaders sometimes do not allow sensitization or awareness on the subject of family planning which affects its uptake in communities. According to her: *“…when these people come to the center, to the facility, we have already conquered the challenge. But the challenges that we encounter are in the community; because if you want to talk with them, the religious leaders around may not allow you to talk about family planning…”* [LGA health official 3, F].

### Achievements in the implementation of the strategic framework for adolescent reproductive health

Some achievements that participants claimed to have resulted from the use of the strategic framework include the increased capacity of AYP to freely discuss matters about sexuality. This is said to be derived from knowledge gained from ASRH health education with concomitant improvement in contraceptive uptake by AYP. However, some of the participants felt that with all these achievements, the State has only been able to implement parts of the policy. A senior Ministry of Health official said: “*so for this policy, I can’t put a mark on the policy but if I can grade it, I will say that we are almost 50% part of what we need to do in terms of this policy implementation* [Ministry of Health Official 5, M]*.* An LGA-level health official was less optimistic saying*: “…looking at the State as a whole there is so much to do and … on a scale of 1-10 maybe I’ll say 2 or 3 in terms of success; That is why I said there is still much to do”* [LGA health official 2, M].

### Gaps in current ASRH policies/activities

One of the gaps we found in this study was the issue of transfer of trained staff, or retirement of trained staff without adequate replacement with skilled and experienced staff members. Similarly, there was reported to be insufficient stepping down of training to younger staff, while most of the experienced staff are on their way to retirement from service. A respondent also said that the current programs and activities were not in all local government areas of the State. According to him*: “…we still need to cascade for the existing program too, it is not in all the LGAs so that means we are still missing out a lot…”* [Ministry of health official 2, M]*.* Some felt that most of the ASRH activities were majorly happening in the urban areas, while the rural areas were left out. For example, one of the youth representatives said: *“…information have still not got to some rural areas, some communities where young people need to know that if they have unprotected sex, there is every tendency that they can have transmission* (of infections)”. A respondent also said that there has been some difficulty in reaching youths that are physically challenged, though the government was working on bridging this gap. He said: *“we’re just bring that* (matters concerning the physcally challenged) *to the fore. For some of our programming, it has been a bit deficient… but we are just trying to bring that on board; reaching out to physically challenged youth has been an issue for us but it’s something we can do moving forward”* [Ministry of health official, 5, M].

Furthermore, some participants felt that data capturing was another gap faced in the implementation of policies and services. This included capturing data from the informal sector such as patent and proprietary medicine vendors (PPMV) and community pharmacies that are used by many young people. For example, a participant said: *“to get data for PPMV has actually been very challenging because they are doing their businesses and costumers walk-in; it is not a project environment, it’s actually a business environment and the first thing the PPMV wants to do is to sell drugs not necessarily to provide some level of data capturing or data management”*[NGO Staff 2, M]*.* Concerning the sustainability of the gains, some participants felt that many of the ongoing interventions were at risk because many are externally funded. A state ministry of health official said: *“…sustainability issues* (remain) *because the programs are being supported by the partners”* [Ministry of health official 2, F]*.* Another said: *“…majorly we need more funding so that the programs can move on because we have a lot of plans. We have a lot of activities but because there is no fund, there is no how we can carry on with those activities that we planned for, funding is the major issue”* [Ministry of health official 1, F].

### Opportunities for improvement of ASRH response

#### Source of funding

While contraceptives were said to be free to AYP in public health facilities, treatment of sexually transmitted infections (STI) is not. An opportunity to have high-level engagement within the State to make STI treatment free to AYP was said to have been identified. A State-level Ministry of Health official said: *“currently we are looking at sending a memo to the Commissioner (of Health), we want to request so that we get these drugs, and youths can easily access these drugs free of charge in our various facilities* [Ministry of Health Official 3, F]*.* A possible source of funding that has been identified is the basic health care provision fund (BHCPF) which is a fund provided for in the National Health Law that States meeting certain criteria can access. According to a member of the ASRH TWG: “… *in the (*implementation of the*) basic health care provision funds, there is a minimum package for the youths, and the state is readily involved and already sponsoring that through the primary health care board, because we have the basic provision fund for family planning …”* [TWG member 2, M]. This may also serve as a basis for creating a separate budget line for ASRH.

#### Political will for promoting the health of adolescent

There is political will for promoting the health of adolescents in the State that stakeholders can leverage on. According to a state-level ministry of health participant: “*we have a good commitment from the government of Ogun state, the honorable commissioner is passionate about maternal and child health being an obstetrician and has committed to investment in adolescent, women and children’s health generally…”* [Ministry of Health Official 3, F]*.*

#### More opportunities for youth-focused NGOs

Given a now favourable political and policy landscape within the state, there is an opportunity for local and international NGOs to invest in ASRH within the state. One of the participants said: *“… we need support from maybe more NGOs, they can come in, support adolescent programs in the State”* [Ministry of Health Official 1, F]*.* Even then, it was emphasized that proper coordination was required to make the most of such a partnership; According to one of the participants: *“…they need to come together … for proper program coordination … so that everybody is not working and doing things on their own haphazardly and we don’t know what implementation they have done…”* [Youth representative 1, F]*.* This will also require the harmonization of work plans across all government agencies implementing youth-focused programs that all stakeholders and partners can buy into and operate within.

#### Integrating research into the policy implementation process

A respondent recommended that adequate feasibility assessments should be carried out and that it was critical that stakeholders/partners conduct needs assessments before the introduction of interventions. He said: *“I think going to school; this thing starts from secondary school… going to school to interview this people, then we can go to market; because these are areas where we have mainly adolescents; … so we need to go to them, interview them, to know what is in their mind, the knowledge they have before we come in with intervention”* [Local government health official 2, F]*.* Such will help in being more effective in meeting the real needs of AYP people rather than assuming what their needs are. Also, none of the activities for ASRH seem to have direct involvement of the academic institutes within the State, as no mention was made of such involvement by the study participants. There is an opportunity here for academics to be a part of the program which will help to drive a research-based policy implementation process.

#### Introduction of other aspects of adolescent health

One of the respondents recommended that there should be an increasing focus on other aspects of adolescents’ and young people’s health including their mental health, and substance use, among others as contained in the strategic framework. He said: *“*[there is a need to also] *concentrate …on the mental health … because the majority of the theme* [here] *is adolescent sexual reproductive health, they are not looking at the mental and psychological health, so I will like … further study to look more at the psychological aspect of the youth and their development.* [Ministry of Health Official 5, M]*.*

## Discussion

The large population of AYP in many developing countries like Nigeria make them to be important targets for health interventions. Supporting their transition into healthy adulthood must continue to be a policy priority. According to an adolescent health and development situation analysis conducted by the Nigeria Federal Ministry of Health in 2018 [11], “*Most States have appointed adolescent health and development (AHD) desk officers in the ministries of health but with no program direction, plan and budget, there is very little that they can do. Except Lagos state”.* The implementation of a strategic framework for adolescent health in Ogun State is changing this narrative. The other States in the country need to take the cue to develop their strategy. However, the promoters in the various states must seek favourable policy windows to ensure successful uptake of the adolescent health-oriented policies just as was done in Ogun State.

Findings from this study indicate that health workers continue to have a negative attitude towards adolescent and young people’s sexual and reproductive health. Mchome et al [25] similarly showed healthcare workers having paternalistic/maternalistic attitudes while lacking knowledge about ASRH services. This discourages young people from using services such as condoms and family planning methods. Some of the challenges that need to be addressed include knowledge of health workers about existing adolescent health policies, competency regarding counseling and interpersonal communication, conflicting personal feelings, cultural and religious values, and beliefs concerning ASRH [14, 25, 26]. Approaches to reducing the negative attitude of health workers to sexual and reproductive health issues of AYP lie in training and retraining of health workers. These should include updating pre-service curriculum, in-service training of all cadres of health workers, and retraining from time to time, on contemporary adolescent sexual and reproductive health issues. This competency-based education in adolescent-responsive health-care is what the World Health Organization (WHO) supports through its “Core competencies in adolescent health and development for primary care providers” document [27]. The document also includes a tool to assess the adolescent health and development component in pre-service education of health-care providers. Given the cultural context within which ASRH exists in many lower income countries, desensitization to ASRH issues among health workers may also be needed to make them less judgmental, and more accepting and understanding of AYP and their peculiarities. Institutionalizing adolescent responsive elements into existing contraceptive services has been successful at a high impact level by making the services responsive to the needs and preferences of adolescents and young people [28].

The findings that there is some level of inclusion of AYP living with disabilities in ASTH programming is welcome and extremely important. The 2018 Nigeria Demographic and Health Survey [29] indicates that up to 7% of household members older than five have some difficulty in at least one functional domain while at least 1% cannot function independently or have a lot of difficulty in doing so. Persons with disabilities suffer from the inability to access basic services, including health care, along with discrimination and stigmatization mostly because of absent, weak, or inadequately executed inclusive policies [30]. This is likely to be even more acute among AYP living with disabilities. It was reported in this study that it was challenging to locate some of these vulnerable people in order to include them in interventions. However, the ongoing efforts must continue. Adolescent health policy implementation must be further strengthened to provide the enabling atmosphere for the protection of these more vulnerable AYP [31]. Such efforts must also include involving young people, including those living with disabilities, in the design, implementation, and evaluation of the AYP SRH programs [32].

It is a critical finding that the coordination of activities of donors/partners working in the adolescent health space is insufficient. This pattern of lack of donor/partner coordination is also demonstrated in the study by Makinde et al. [33] where between 2010 and 2016, there were ten different donor-funded health facilities listing efforts within the country, indicating duplication of efforts. The increased number of donors focusing on one area has led to duplication of efforts, and concentration of intervention [33, 34]. The best approach to managing donor activities perhaps is to design a coordination mechanism [35]. Also, without coordination, competition among donors may shift their focus away from the business of providing aid to a fight for market recognition [36]. This is one of the reasons a technical working group at the state level is always necessary, which will be tasked with creating a platform for all stakeholders to interact. Coordinating activities can include mapping of all partners, their areas of activities (including geographical location), and compulsory registration of all interventions being carried out by any partner. The mapping will also help to avoid program concentration in a few places and/or overlap of efforts.

Another gap we found in this study is the missed opportunity to strengthen policy implementation with research. Activities for ASRH in the State didn’t seem to involve academic institutes within the state. A likely reason for non-involvement of academic institutes in ASRH within the state is because many programs are supported by partners who usually have their compliments of researchers/consultants, often from outside the location of intervention. This is partly demonstrated by the eligible participant in this study who declined participations because of organizational policies. The challenge in this setup is that local research capacity is not retained within the state to meet future needs after the funded program has ended. To bridge such gaps, the academic institutes must also be proactive in seeking a ‘seat at the table’. Researchers in Ebonyi State, Nigeria started the Health Policy Advisory Committee (HPAC), as a health policy advisory committee to facilitate knowledge translation through capacity building, mentorship, and stakeholder policy dialogues [37]. This effort was reported to have promoted cooperation between policy-makers and researchers. This lays credence to the argument of Hawkes et al. [38] that evidence-informed policy can be sustainable when institutional capacity and proper management of the political environment occurs. There is an opportunity in Ogun State to institute a viable research-based policy implementation process if these recommendations are followed.

There seems to be a political will for promoting the health of adolescents in the Ogun State that stakeholders can leverage on. Presence of political will is a window of opportunity to leverage funding for adolescent health services through the internal funding called the Basic Health Care Provisions Fund (BHCPF). The BHCPF is a statutory allocation of not less than 1% (revised to 2% following revision of the law in 2022) of the Consolidated Revenue Fund (CRF) – that is, the total Federal Revenue before it is shared with all tiers of government [39]. It is to be used to provide a basic package of services in primary health care facilities through the National Health Insurance Scheme (NHIS), the National Primary Health Care Development Agency (NPHCDA), and the Federal Ministry of Health. Its contribution of 25% in counterpart-funding towards specified primary health care (PHC) projects could be very useful in improving health services for young people. Policy stakeholders should use advocacy and communication to make a case for funding adolescent health through this internal fund.

Sustainability is always a challenge when programs are externally funded by donors. The World Health Organization defines sustainability as the ability of a project to continue to function effectively, with high treatment coverage, and integrated into available health care services, with community ownership, and community and governmental funding using resources mobilized by the community and government [40]. Often, lack of funding is the most critical threat to sustainability. Policy stakeholders must be innovative and creative in addressing these funding challenges. In addition to the BHCPF already identified, there may be local funders interested in promoting these services. We recommend that policymakers within the state should actively seek out funders, for instance, manufacturers of menstrual sanitary products. Some of these factories are located within Ogun State, and are easily accessible [41].

One of the recommendations provided by a participant in this study was the need for increased focus on other aspects of adolescent and young people’s health including their mental health, and substance use, among others. Looking into the future, SRH of adolescents should be used as a springboard for mainstreaming other aspects of adolescent health. Taking an integrated approach to the health of adolescents and young people can enable such a procedure [42]. While this integrated approach is enshrined in the Ogun State adolescent reproductive health strategic framework, its implementation was less evident.

### Limitation

The findings in this study are based on the opinions and experiences of the key informants interviewed. While the level of agreement among the different participants suggests reliability, it is important to indicate that the majority were, or are, direct or indirect participants in the implementation of the strategic framework for adolescent reproductive health. It is equally possible that some participants gave socially desirable responses since they may want to paint a favourable picture of ASRH responses in the State. However, during data collection, the participants were reminded to be as truthful and factual as possible in the infomation they provided.

### Implications for practice, policy, and research

**Fig. 2:**
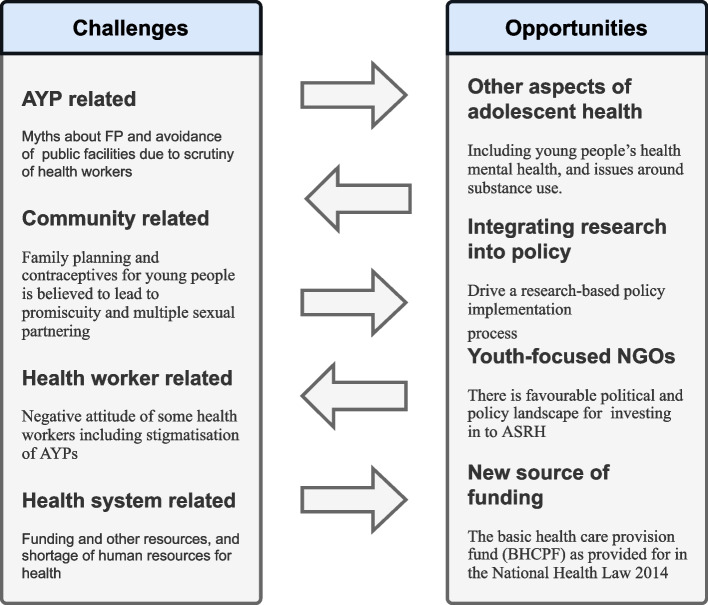
Policy- and service-level challenges and opportunities in implementing SRH services for AYP in public health facilities in Ogun State, Nigeria

The strategic framework for adolescent reproductive health being used in Ogun State can serve as a template that other States can adopt for scaling up their programs for AYP. Also, the Basic Health Care Provision Fund is a potential source of funding for adolescent health but there are usually political ramifications to such sources of funding. Policymakers and other stakeholders will need to muster all their advocacy abilities and be on the lookout for favourable policy windows such as a sympathetic political leader or spouse of a political leader. They must push for a more permanent institutionalization of ASRH response in the state since the tenure of the current framework will elapse in 2022. There may be the need for expanding the scope of interventions and programs to include specific programs that target parents with health education. Parents/guardian sometimes constitute part of the sociocultural barrier to accessing sexual and reproductive health services by AYP. However, they can be co-opted as partners-in-progress by helping them to come to the understanding of relevant issues in adolescent reproductive health, and showing them ways their AYP can be kept from adverse health outcomes. Finally, there is the need to continue to provide the AYP with the correct information about contraceptives and family planning. Many have misconceptions about this but high rates of teenage pregnancy continues to be a challenge [43]. The key challenges and opportunities in implementing SRH services for AYP found in this study are summarized in Fig. 2. Future research is needed for understanding the priorities and preferences of AYP to be able to design services that optimally meet their SRH needs.

## Conclusion

This study provided the context of the implementation of the strategic framework for adolescent reproductive health in a Nigerian setting from the perspectives of policy- and service-level stakeholders. The findings indicate the need for continued implementation of adolescent-responsive contraceptive services, increasing the reach of programs to adolescents living with disabilities, stronger coordination among partners, and leveraging new sources of funding. Opportunities for improving program delivery identified include ensuring research-based policy implementation and seeking program sustainability through tapping into internal funding such as the Basic Health Care Provisions Funds (BHCPF).

## Data Availability

The datasets used and/or analysed during the study are available from the corresponding author on reasonable request.
